# The Usage of Ergot (*Claviceps purpurea* (fr.) Tul.) in Obstetrics and Gynecology: A Historical Perspective

**DOI:** 10.3390/toxins13070492

**Published:** 2021-07-15

**Authors:** Aleksander Smakosz, Wiktoria Kurzyna, Michał Rudko, Mateusz Dąsal

**Affiliations:** 1 Department of Pharmaceutical Biology and Biotechnology, Faculty of Pharmacy, Wroclaw Medical University, 50-367 Wroclaw, Poland; aleksander.smakosz@gmail.com; 2 Department of Humanities and Social Science, Faculty of Pharmacy, Wroclaw Medical University, 50-367 Wroclaw, Poland; wiktoria.kurzyna@student.umed.wroc.pl; 3 Department of Physical Chemistry, Faculty of Pharmacy, Wroclaw Medical University, 50-367 Wroclaw, Poland; michalzdzislawrudko@gmail.com

**Keywords:** ergot, ethnopharmacology, abortion, childbirth

## Abstract

In the past centuries consumption of bread made of ergot-infected flour resulted in mass poisonings and miscarriages. The reason was the sclerotia of *Claviceps purpurea* (Fr.) Tul.—a source of noxious ergot alkaloids (ergotamine and ergovaline). The authors have searched the 19th century medical literature in order to find information on the following topics: dosage forms of drugs based on ergot and their application in official gynecology and obstetrics. The authors also briefly address the relevant data from the previous periods as well as the 20th century research on ergot. The research resulted in a conclusion that applications of ergot in gynecology and obstetrics in the 19th century were limited to controlling excessive uterine bleeding and irregular spasms, treatment of fibrous tumors of the uterus, and prevention of miscarriage, abortion, and amenorrhoea. The most common dosage forms mentioned in the works included in our review were the following: tinctures, water extracts (Wernich’s and Squibb’s watery extract of ergot), pills, and powders. The information documented in this paper will be helpful for further research and helpful in broadening the understanding of the historical application of the described controversial crude drugs. Ergot alkaloids were widely used in obstetrics, but in modern times they are not used in developed countries anymore. They may, however, play a significant role in developing countries where, in some cases, they can be used as an anti-hemorrhage agent during labor.

## 1. Introduction

In the history of mankind, *materia medica* representatives have experienced their ups and downs. Plant and animal raw materials have been commonly used in medicine, pharmacy, and other economic sectors. However, fungal raw materials were usually out of the scope of interest. On the other hand, it is difficult not to appreciate the significance of *Saccharomyces cerevisiae* (Desm.) Meyen ex E.C. Hansen (brewer’s yeast and baking yeast; with biotechnology, it is possible to produce various drugs with cultures of the above species).

The situation is a bit different with sclerotia of *Claviceps purpurea* (Fr.) Tul. (ergot)—a source of unknown origins and unknown ancient history. Through the ages, this medicinal fungus has been closely connected with gynecology and obstetrics.

We do not know if the ancients could identify and cultivate rye. Possibly this species was used in Thrace and Macedonia to bake bread. Dated around 600 BC, an Assyrian tablet alluded to a “noxious pustule in the ear of grain” (Could it be ergot?). In classical Latin, rye was called secale [1]. A little later, during the medieval period, it was called siligo [1].

A large number of *Poaceae* representatives may become infected by fungi belonging to the *Claviceps* genus [1,2]. However, from both the historical and economical point of view, the most important one is *Claviceps purpurea* (ergot), which causes damage to rye, wheat, and barley [2].

In the past centuries, consumption of bread made of ergot-infected flour resulted in mass poisonings. With regard to symptoms, two forms of ergot poisoning can be distinguished—*ergotismus gangrenosus* and *ergotismus convulsivus* [3]. The first stage of the poisoning is similar in both cases—gastrointestinal and abnormal crawling sensation in the limbs which later develops into pain. With time, the poisoning develops into one of the types mentioned above. In the case of gangrenous ergotism, ischemia affecting the limbs results in distal changes of skin color and then gangrene, which in turn causes the loss of limbs or even death [3]. Convulsive ergotism manifests itself in nervous system disorders. This form of poisoning causes painful involuntary muscle twitching while the body of the person affected by the illness takes abnormal postures. In some cases, mania and hallucinations occurs simultaneously [2,3].

*Ergotismus gangrenosus* is most probably an equivalent of the so called “St. Anthony’s Fire”, while *ergotismus convulsivus* is sometimes compared to “St. Vitus Dance” [1,2,3]. However, the majority of sources associate it with Huntington’s disease [3]. The medieval epidemics of *ergotismus gangrenosus* were common in the regions of Europe west of the Rhine (France), while *ergotismus convulsivus* occurred mostly in the part of Europe east of the Rhine (Germany) and in Scandinavia [3,4]. Ergotism epidemics frequently occurred in communities where the diet was rich in rye and took place after cold and wet winters followed by wet springs, as high air moisture and wind facilitate the spreading of the *Claviceps purpurea* fungus. It was mentioned that breast-fed infants did not show any poisoning symptoms [3].

The last time ergot poisoning happened in Europe on a larger scale was in Westphalia, Hanover, and Lauenburg in 1771 where in some villages only 5 out of 120 people survived [3]. It is also implied that ergot may be responsible for the well-known “choreomania” (dancing plague) during which European villagers of the Middle Ages were falling into an involuntary dance trance [3].

## 2. Ethnomycology of *C. purpurea*

### 2.1. Nomenclature of Ergot

For centuries, many scientists had difficulties with classifying ergot. In 1816, Augustin Pyramus de Candolle (Swiss botanist who specialized in economic botany and agronomy) described *Secale Cornutum* as a fungus and named this species *Sclerotium Clavis* [5]. Another Swiss researcher—Elias Magnus Fries (professor of botany and applied economics at Uppsala University, the father of modern fungal taxonomy)—denominated it *Spermoëdia Clavus* [5]. What is crucial is that Edwin John Queckett (botanist, surgeon, and microscopist) called it *Ergotoetia abortifaciens* (*abortifaciens*—Latin term for a substance that induces abortion). In 1839 he wrote [6] the following: “I adopted the term abortans […] is it probable that I ever should have proposed […] name as being fitted to form the specific one of the newly discovered genus.”

Miles Joseph Berkley (Anglican clergyman interested in plant pathology) was also conscious of the specific effect of the aforementioned fungus and therefore he called it *Oidium abortifaciens* [5]. In 1853, Edmond Tulasne (French mycologist and botanist) introduced his views on the development cycle of ergot. Since then, this fungus has been called *Claviceps purpurea* [5].

This name, however, was not widely accepted and editors of contemporaneous London Pharmacopoeia referred to ergot as *Acinula Clavus*—a species never described before [2]. In pharmaceutical sources, ergot was called *Clavus siliginis, Calcar, Secalis mater*, *Secale luxurians*, *Secale cornutum*, and *Grana secalis degenerate* [2,4].

### 2.2. Ethnopharmacology of C. purpurea

We have no clear evidence as to when ergot was first introduced into medicinal use in the West. However, its history in the East is clearer. Alexander Tschirch (pharmacist and pharmacognosist who lived at the turn of the 20th century) stated that Chou Kung (Chinese philosopher and physician) wrote about ergot circa 1100 BC [1]. According to this man of science, this source was used as an obstetrical remedy—the application was unknown in Europe until the 17th or 18th century. On the other hand, the further works associated with botany, e.g., “Thousand Golden Remedies” of Sun Simiao written in the 7th century and “Pen Ts’ao Kang Mu” written in the 16th century by Li Shih-chen (he complied 11,091 prescriptions used throughout centuries; these are listed in 52 volumes describing 443 animal substances and 1,074 plant materials) do not mention ergot [1].

Rye was primarily cultivated by the Teutons who were a Germanic tribe [1], so it should not be surprising that the first medicinal reference to ergot as a drug comes from German sources. Adam Lonicer was a German botanist and physician in the city of Frankfurt [7]. He was an author of the *Kreuterbuch*—one of the most notable herbals in the history of herbalism and pharmacy. In 1582, the fourth edition of this work was published. In the chapter concerning agricultural crops and grains (the subchapter devoted to rye), he wrote that sometimes long black grains stick out of the spike similar to long nails [7]. This description clearly indicates that Lonicer knew what ergot was. Furthermore, he also wrote that women used three sclerotia of the *Claviceps* to induce a strong uterine contraction [7]. This is the oldest evidence of *Secale cornutum* application in gynecology and obstetrics.

The oldest known illustration (woodcut) of ergot can be found in a book entitled the following: “Botanical theatre or the history of plants” (lat. *Theatrum Botanicum Sive Historia Plantarum*) edited by the son of Caspar Bauhin in 1658 [8].

## 3. Ups and Downs of Ergot Application in Gynecology and Obstetrics

### 3.1. Excessive Uterine Bleeding and Irregular Spasms

According to John Stearns the official usage of ergot in gynecology started in 1747 in Holland. It was subsequently used in France until it was interdicted in 1774 by a legislative act [9]. In the first decade of the 19th century in the state of Washington, there was a high-profile case of a Scottish woman who applied ergot medications in obstetrics practice with fatal results. These issues were associated with the overdosage of C. purpurea. In addition, this fungal resource is very variable in its active constituent content. Due to this quality, it is highly unlikely to determine what a safe dose should be. Dr. J. Stearns, a physician from the State of New York, began applying ergot-based drugs in his gynecology practice in 1807 [9]. Due to the of the rejection of this drug by previous generations, he had to collect it by hand. He started with ergot powder and then he tried the decoction, which he considered superior. The maximum dose was 10 grains (an equivalent of about 32.4 g) but the regular dose was much lower [9]. In the following years, in the USA, a clinical trial net of obstetricians who used ergot in their practice in form of powder and decoction was established [9]. Stearns called this powder “Childbirth powder” (lat. *pulvis parturiens*) [4]. In 1813, the trial results were published in *The New England Journal of Medicine and Surgery*. They were met with general approval. What is important is that some of the physicians described the cases of children that were stillborn after ergot had been applied [9]. This is not surprising—*C. purpurea* is a very potent medication. Due to prior French objections, some American doctors considered the “new” drug to be harmful and worthless [9].

The British doctors were more reserved—they valued the action of ergot, but they observed that application of ergot during pregnancy and delivery was associated with a greater death rate of infants. Apart from applying ergot in midwifery they also tried to use *C. purpurea* in cases of amenorrhoea [4].

The approach to this raw material in France changed in 1872 when the Academy of Medicine in Paris enacted Article 32 of the law of the 19th Ventose and allowed midwives to prescribe ergot [10]. The Academy stated that ergot-based medicines had a lot of advantages in obstetric practice. That regulation was in opposition to the previous laws and decrees, which restricted the prescription of poisons to physicians and veterinarians [10]. Therefore, pharmacies were able to provide gynecological drugs based on sclerotia of *C. purpurea* to a new group of medical professionals.

It is important to point out that usage of ergot was limited to cases that posed a threat to the life of both mother and child. In such cases, the risk was legitimate. Taking this into consideration, Dr. John Stearns created a list of observations regarding the use of ergot [9]. He stated that it should never be administered during labor and in quantities larger than thirty grains (an equivalent of about 48.6 g) [9]. Combined with opium and water, the sclerotia of *C. purpurea* were given when interrupted pains of regular labor occurred (dosage: teaspoonful was administered every ten min). The described drug was contraindicated when regular labor or the contractions were uninterrupted [9].

On the other hand, application of ergot was indicated in the case of extending labor when the contractions were irregular and/or too weak to advance the labor or when the contractions and pains were transferred from the uterus to the other parts of the body, giving puerperal convulsions [9].

In 1814, Dr. Henry S. Waterhouse witnessed difficulties during childbirth [9]. The patient reported vaginal bleeding and lower abdomen constriction. Then, she began to lose consciousness and started bite her tongue. In the hours that followed, the doctor began to observe alarming contractions of the muscles of her limbs, back, abdomen, neck, and lower jaw [11]. Conventional drugs of the time such as tincture of opium and tincture of asafoetida (*Ferula assa-foetida* L.) had not stopped the symptoms from progressing [9]. Eventually, he decided to give her a mixture of 30 grains of ergot with water. The administration of this medication stopped the worsening of her condition and she was soon able to deliver the baby successfully [9].

Another important remark on ergot was made by Dr. John Paterson who specialized in midwifery. In 1840, he wrote the following [11]: “I consider the ergot more to be depended on, as to its particular effects on the uterus than almost any other specific in the Pharmacopoeia.”

This scientist also mentioned that he had never seen any side effects of ergot application. It is rather implausible since just a single dose can cause symptoms such as vomiting, colic, pains, headache, and hallucinations [9,11].

Considering the side effects mentioned above, many pharmacists, physicians, and herbalists tried to compound an ergot-based drug with as few side effects as possible. Dr. Rees’s ethereal solution seemed to be the best choice. According to *The Retrospect of Practical Medicine and Surgery* from 1840 [11]: “The ethereal solution, the properties of which you have so well tested, was prepared by digesting 4 ounces. of the powdered ergot in 4 fluid ounces of ether for seven days. The result was a solution of the fatty matters contained in the drug: this was poured off, evaporated to dryness, and the residue again dissolved in 2 fluid ounces of ether.”

One part of the preparation was the equivalent of two parts of ergot. Both ether and Claviceps possess a narcotic potential and so usage of the ethereal solution was highly hazardous [11].

Other forms of ergot-based drugs used in obstetrics practice were emulsions, mucilages, syrups, and water extracts mixed with aromatic water [2].

### 3.2. Fibrous Tumor of the Uterus

In the 19th century, treatment of a fibrous tumor of the uterus was regarded as beyond the reach of medicine. One of the most problematic symptoms associated with this illness is excessive uterine bleeding and hypertrophy. Ergot-based medicines worked via elimination (excretion) of polyp or intramural tumor [12]. Dr. Byford used a fluid extract of ergot in this treatment (half spoonful for three weeks; both by mouth and sometimes hypodermically). As a result of the therapy, the tumor was expelled and the uterus inverted. The surgeon had to remove the remaining fibroids. In some cases, the pain during therapy was intolerable and therefore the treatment had to be terminated before obtaining expected therapeutic effect. At that time, there were two main compounds used in the therapy of fibrous tumors of the uterus—Wernich’s watery extract of ergot and Squibb’s watery extract of ergot [12,13].

The first one was yielded by the extraction of the drug: First with ether, then with ethanol, and lastly with water; then the solvent evaporated. Squibb’s compound was a simple water extract evaporated on an evaporating dish [13,14]. Both drugs were mixed with glycerin and sometimes with belladonna (*Atropa belladonna* L.) or opium [12,15]. The therapy described above was forgotten until 1953, when further studies on the effects of pure ergometrine on this type of cancer (especially associated with uterine bleeding) were conducted [14]. In this case, the dose of 0.2–0.4 mg of ergometrine was used [14].

### 3.3. Abortion and Poisoning

In 1844, there was a trial of a physician charged with the intention to procure an abortion [16]. The accuser held that Dr. James Calder administered noxious pills made of powdered savin juniper and essential oil (*Juniperus sabina* L.) and a decoction of ergot to an unmarried woman. In addition, he gave her powder compounded of iron (II) carbonate and cantharides (*Lytta vesicatoria* L.) [16]. In the further proceedings, it turned out that they were in a relationship and she was likely impregnated by him [16]. Therefore, he tried to induce an abortion by instructing her to take two pills a day. Due to the terrible smell and taste, she threw them into the fireplace [16].

Another example of a similar case took place in 1889 [17]. The prosecutrix—a nurse at the Selby Oak Workhouse—accused a surgeon named Cuthbert of an intentional abortion procured with the fluid extract of ergot. Due to unreliable evidence, the trial ended without a sentence. This result may have been influenced by the support of the entire medical community [17].

Poisoning (both criminal and suicidal) with ergot was (and is) uncommon. Cases in which the autopsy proved death by poisoning with this crude drug were usually connected with an attempt to procure abortion. One such case was reported in 1864 [18]. The victim—a young unmarried woman—had all the clinical signs of ergot poisoning before her death—yellow skin, vomiting, headache, dryness, and irritation of the throat, and intestine hyperemia [18]. Death occurred due to the aforementioned *ergotismus gangrenosus*. Further investigation demonstrated that she had been consuming the tincture or ergot and pennyroyal (*Mentha pulegium* L.) essential oil for 11 weeks [18]. The latter substance was used as an abortifacient from the times of Ancient Greece [19].

On the other hand, the administration of ergot in the case of profuse hemorrhage could prevent miscarriage. This kind of treatment was suggested by Dr. A. Freer who declared that he used this medicine in such cases over 200 times [20]. At the same time, he wrote, with a high level of uncertainty, about the application of ergot in abortion (10th–12th week) [20]. Some practicing physicians had the opposite experience with this remedy. Dr. John Basset published his remarks on the application of ergot as a medical abortion drug in 1872 [21]. He expressed the opinion that contractions of the muscular fiber (caused by a pulverized sclerotia) are too weak to expel the fetus. Moreover, the ergot alkaloids stopped uterine bleeding—which is a significant occurrence during the abortions performed at that time [21].

According to D. Allen and G. Hatfield (ethnobotanists), only a solitary and contemporary record of folk usage of ergot in procuring abortions (in Norfolk) has been traced, but it may have been as well a widespread practice throughout the centuries [22].

It is necessary to notice that ergot-induced poisoning is also an issue in animal husbandry. Cattle (*Bos taurus* L.) are very sensitive to ergot alkaloids (especially ergotamine, ergovaline, ergonovine, ergocristine, and ergocornine). Clinical signs of ergotism in cattle include convulsions, ataxia, gangrenous extremities, vasoconstriction, and abortion [23].

## 4. Contemporary Pharmacology of Ergot

As stated above, ergot was used as a crude drug until the 19th century. In the 20th century, individual alkaloids were extracted and described. Ergotoxine was discovered and described in 1906 as a single chemical compound. This state of affairs held until 1943, when individual alkaloids were isolated from it. Another alkaloid, ergotamine, was isolated in 1918 by Stoll. Lysergic and isolysergic acids were described in 1934 and 1936, respectively. In 1935, another alkaloid was discovered—ergometrine [24,25].

**Figure 1:**
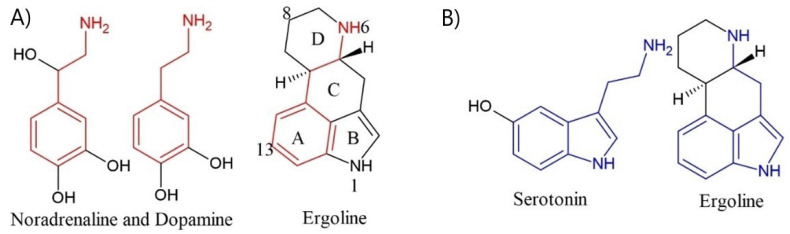
Comparison of dopamine, noradrenaline, and serotonin structure with ergoline. Similarities are exposed by utilizing color (**A**) dopamine, noradrenaline and ergoline; (**B**) Serotonin and ergoline.

Ergoline is a core chemical structure of ergot alkaloids. The molecular mechanism of drug action is associated with the impact of ergot alkaloids on dopamine, noradrenaline, and serotonin receptors via structural similarities between aforementioned molecules and ergoline core [26]. Similarities of molecular structure are shown in Figure 1.

By modifying the lysergic acid, a range of derivatives with different receptor activity could be obtained. Amide derivatives of lysergic acid with a small side chain have less adremolytic and higher 5HT antagonist activity. Hydrogenation of ergotamine class alkaloids results in a higher adrenolitic effect [27]. Nicergoline is one of the examples of hydrogenation impact on ergot alkaloids derivatives as it is administered in hypertension strong α_1_-receptor blocker [26]. The introduction of elaborate moieties in C-13 or C-14 has a strong impact on weakening the interaction with dopamine receptors and results in increased selectivity towards 5-HT_2_ receptors. Selectivity towards 5-HT_1_ receptors can be obtained via modification of the C and D ring of ergoline core [27].

Ergotamine and dihydroergotamine are α-adrenergic agonists/antagonists, display dopamine 2 receptors agonistic activity, and show partial agonist action on 5-HT receptors. Ergometrine is an α-adrenergic partial agonist with no impact on dopamine 2 receptors and has partial agonist action on 5HT receptors [27,28].

Ergot alkaloids in obstetrics are administered at the third stage of labor to prevent postpartum hemorrhage. They must be administered with great caution because of side effects, e.g., blood pressure elevation and pain. Thus, the dose must be chosen with great care because of the side effects. Employment of ergot alkaloids is more important in developing countries where postpartum hemorrhage is the cause of many deaths and where access to modern medicine methods is limited [28]. On the other hand, the administration of oxytocin in postpartum hemorrhage prevention (in contrast to ergot alkaloids) can result in a prolonged third stage of labor [29]. Ergot alkaloids used to be widely applied in obstetrics and yet, nowadays, only a few are still used. Ethylergonovine is used as a highly effective second-line uterotonic medication (unfortunately it is associated with severe vasoconstriction) [30]. In the developing countries in some cases, ergot alkaloids can be the only anti-hemorrhage agents available during labor. The summary of the ergot alkaloids properties is shown in Table 1.

## 5. Conclusions

In conclusion, the application of ergot in gynecology and obstetrics in the 19th century was limited to controlling excessive uterine bleeding and irregular contractions, treatment of fibrous tumors of the uterus, and prevention of miscarriage. There is little evidence that sclerotia of the *Claviceps purpurea* were used in abortion or amenorrhoea. In the cases described above, the aforementioned abortifacient was either ineffective or caused deaths of the patients. On the other hand, abortion in in the 19th century was in most cases illegal and so information about the usage of abortive medications was a type of taboo.

The most common dosage forms mentioned in the works and that are included in our review were the following: tinctures, water extracts (Wernich’s watery extract of ergot, Squibb’s watery extract of ergot), pills, and powders. The information documented in this paper will be helpful for further research and for broadening the understanding of the historical application of the described controversial crude drug. Twentieth and twenty-first century applications of ergot in medicine will be covered by the authors in future papers.

## 6. Methodology

The authors conducted a literature search within the JSTO [35] and archive.org [36] databases using keywords such as “Ergot obstetrics”, “Ergot abortion”, and “Ergot gynecology” between 1822 and 1896 (published in the United Kingdom and the United States of America). After reading the works, we selected the most relevant papers and books for this article. Regarding the inclusion criteria, the works were selected depending on the inclusion based on the following topics: dosage forms of drugs based on ergot and their application in official gynecology and obstetrics. Exclusion criteria include the following: All other articles that did not cover one of these topics as their primary endpoint. The binominal Latin names of plants were synchronized with The Plantlist [37] database and binominal names of fungi were synchronized with the Index Fungorum [38] database.

## Figures and Tables

**Table 1:** Active compounds found in ergot, application, year of discovery, and impact on receptors for chosen compounds.

Compound	Action/Application	Year of Discovery	Refs.
Ergotamine	Uterotonic, acceleration of labor, restriction of postpartum hemorrhage	1918	[24,26]
Dihydroergotamine	Sympatholytic, shortening labor.	1943 ***	[26,31,32]
Lysergic acid	Basic compound for further synthesis.	1934	[24]
Isolysergic acid	Basic compound for further synthesis.	1936	[24]
Ergometrine (also named ergonovine)	Uterotonic, restriction of postpartum hemorrhage	1935 **	[25,26]
Ergotoxine	Restriction of postpartum hemorrhage	1906, 1943 *	[24]
Ergocristine	No significant use in obstetrics	1943 *	[24]
Ergokryptine	Lactation suppresion, shortening of gestation period in animal model.	1943 *	[24,28,33]
Ergocornine	Suppression of prolactine secretion.	1943 *	[24,29,34]

* Discovery that ergotoxine is a mixture of alkaloids, ** extraction and characterization, *** year of the first synthesis, Ref. = Reference.

## References

[1] Bove F.J. (1970). The Story of Ergot.

[2] Christison R. (1848). A Dispensatory or Commentary on the Pharmacopoeias of Great Britain.

[3] Eadie M.J. (2003). Convulsive ergotism: Epidemics of the serotonin syndrome?. Lancet Neurol..

[4] Presscot O. (1813). A Dissertation on the Natural History and Medicinal Effects of Secale Cornutum, or Ergot.

[5] Felter H.W., Lloyd J.U. (1905). King’s American Dispensatory.

[6] Queckett E.J. (1839). Origin of the ergot of rye. Lancet.

[7] Lonicer A. (1840). Kreuterbuch.

[8] Bauhin C. (1658). Theatrum Botanicum Sive Historia Plantarum.

[9] Stearns J. (1822). Observations on the Secale Cornutum, or Ergot; with Directions for its use in Parturition. Am. Med. Rec..

[10] (1872). Prescription of ergot by midwives. Br. Med. J..

[11] Braithwaite W. (1840). The Retrospect of Practical Medicine and Surgery, No. 1..

[12] (1876). The Treatment of Fibrous Tumours of the Uterus by Ergot. BMJ.

[13] Martin F.H. (1896). Treatment of Uterine Fibroids. JAMA.

[14] Heaney N.S., Hyman E. (1953). Conservative Therapy of Benign Uterine Bleeding—With Special Reference to the Use of Ergot. Calif. Med..

[15] Smart A.R. (1876). Ergot, Its Physiological and Therapeutical Action: Read Before the Southern Michigan Medical Society.

[16] Shapter T. (1844). Report of the Trial of a Medical Practitioner, on a Charge of Intent to Procure Abortion. Prov. Med. Surg. J..

[17] (1889). The Charge of Intent to Procure Abortion at Selby Oak. Lancet.

[18] Stephens J. (1864). The Case of Fatal Attempt to Procure Abortion. BMJ.

[19] Nelson S.E. (2009). Persephone’s Seeds: Abortifacients and Contraceptives in Ancient Greek Medicine and Their Recent Scientific Appraisal. Pharm. Hist..

[20] Freer A. (1872). Ergot in Abortion. BMJ.

[21] Basset J. (1872). Ergot in Abortion. BMJ.

[22] Allen D.E., Hatfield G. (2004). Medicinal Plants in Folk Tradition.

[23] Craig A.M., Klotz J.L., Duringer J.M. (2015). Cases of ergotism in livestock and associated ergot alkaloid concentrations in feed. Front. Chem..

[24] Hofmann A. (1978). Historical view on ergot alkaloids. Pharmacology.

[25] MacLean A.B. (2005). Ergometrine. J. Obs. Gynaecol..

[26] Sharma N., Sharma V., Manikyam H., Krishna A. (2016). Ergot Alkaloids: A Review on Therapeutic Applications. Eur. J. Med. Plants.

[27] Tudzynski P., Correia T., Keller U. (2001). Biotechnology and genetics of ergot alkaloids. Appl. Microbiol. Biotechnol..

[28] Liabsuetrakul T., Choobun T., Peeyananjarassri K., Islam Q.M. (2018). Prophylactic use of ergot alkaloids in the third stage of labor. Cochrane Database Syst. Rev..

[29] Salati J.A., Leathersich S.J., Williams M.J., Cuthbert A., Tolosa J.E. (2019). Prophylactic oxytocin for the third stage of labor to prevent postpartum haemorrhage. Cochrane Database Syst. Rev..

[30] Vallera C., Choi L.O., Cha C.M., Hong R.W. (2017). Uterotonic Medications. Anesthesiol. Clin..

[31] Silberstein S.D., McCrory D.C. (2003). Ergotamine and dihydroergotamine: History, pharmacology, and efficacy. Headache.

[32] Altman S.G., Waltman R., Lubin S., Reynolds S.R.M. (1952). Oxytocic and toxic actions of dihydroergotamine-45. Am. J. Obs. Gynecol..

[33] Whitacre M.D., Threlfall W.R. (1981). Effects of ergocryptine on plasma prolactin, luteinizing hormone, and progesterone in the periparturient sow. Am. J. Vet. Res..

[34] Welsch C.W., Iturri G., Meites J. (1973). Comparative effects of hypophysectomy, ergocornine and ergocornine-reserpine treatments on rat mammary carcinoma. Int. J. Cancer.

[35] JSTOR. https://www.jstor.org/.

[36] Internet Archive. https://www.archive.org/.

[37] The Plant List Version 1.1. https://www.theplantlist.org/.

[38] Index Fungorum. http://www.indexfungorum.org/.

